# Circulating microRNA as biomarkers of canine mammary carcinoma in dogs

**DOI:** 10.1111/jvim.15764

**Published:** 2020-04-27

**Authors:** Eric J. Fish, Esther Gisela Martinez‐Romero, Patricia DeInnocentes, Jey W. Koehler, Nripesh Prasad, Annette N. Smith, Richard Curt Bird

**Affiliations:** ^1^ Department of Pathobiology, College of Veterinary Medicine, AURIC—Auburn University Research Initiative in Cancer Auburn University Auburn Alabama United States; ^2^ Genomic Services Laboratory Hudson Alpha Institute for Biotechnology Huntsville Alabama United States; ^3^ Department of Clinical Sciences, College of Veterinary Medicine Auburn University Auburn Alabama United States

**Keywords:** canine, microRNA, serum biomarkers, mammary carcinoma

## Abstract

**Background:**

Differentiating benign from canine malignant mammary tumors requires invasive surgical biopsy. Circulating microRNAs (miRNA) may represent promising minimally invasive cancer biomarkers in people and animals.

**Objectives:**

To evaluate the serum mRNA profile between dogs with and without mammary carcinoma, and to determine if any of these markers have prognostic significance.

**Animals:**

Ten healthy client‐owned female dogs (5 intact, 5 spayed) and 10 dogs with histologically confirmed mammary carcinoma were included; 9 were client‐owned, whereas 1 was a research colony dog.

**Methods:**

Retrospective study. Serum miRNA was evaluated by RNA deep‐sequencing (RNAseq) and digital droplet PCR (dPCR).Expression of candidate biomarkers miR‐18a, miR‐19b, miR‐29b, miR‐34c, miR‐122, miR‐125a, and miR‐181a was compared with clinical characteristics, including grade, metastasis, and survival.

**Results:**

452 unique serum miRNAs were detected by RNAseq. Sixty‐five individual miRNAs were differentially expressed (>±1.5‐fold) and statistically significant between groups. Serum miR‐19b (*P* = .003) and miR‐125a (*P* < .001) were significantly higher in the mammary carcinoma group by dPCR. Both had high accuracy based on receiver operator characteristic area under the curve (0.930 for miR‐125a; 0.880 for miR‐19b). Circulating miR‐18a by RNAseq was significantly higher in mammary carcinoma dogs with histologic evidence of lymphatic invasion (*P* = 0.03). There was no significant association with any miRNA and survival or inflammatory status.

**Conclusions and Clinical Importance:**

Circulating miRNAs are differentially expressed in dogs with mammary carcinoma. Serum miR‐19b and miR‐18a represent candidate biomarkers for diagnosis and prognosis, respectively.

**Abbreviations**
AUC: area under the curve
CMT: canine mammary tumor(s)
dPCR: digital droplet PCR
FAM: 6‐carboxyfluorescein
miRs/miRNA: microRNA
qPCR: quantitative PCR
RNAseq: RNA deep‐sequencing
ROC: receiver operator characteristic

## 1 INTRODUCTION

Mammary tumors in dogs (canine mammary tumors, CMT) are 1 of the most common neoplasms in sexually intact female dogs, with a variable prognosis reflective of the fact that approximately half are benign and half are malignant.^1^ Key histologic prognostic factors include subtype, grade, stage, and evidence of lymphatic invasion. Dogs with Grade III tumors have significantly shortened survival compared with dogs that have Grade I or II CMT.^2^ Dogs with tumor lymphatic invasion identified in biopsies have a 3‐fold higher rate of tumor recurrence, distant metastasis, and death.^2^ Finally, dogs with inflammatory mammary carcinoma have widespread metastasis and a grave prognosis with average survival of 25 days from diagnosis.^3^, ^4^ Obtaining this prognostic information currently requires an invasive tissue biopsy. A minimally invasive biomarker for CMT could improve detection and clinical decision‐making. Biomarkers are any quantitative measure of a disease or physiological state, and commonly include a variety of traditional or novel biochemical analytes.^5^

Circulating microRNAs (miRNA) are small noncoding RNA molecules present in blood that show promise as powerful noninvasive biomarkers in human oncology. Unlike most RNA, serum miRNA concentrations are stable over time, temperature, and multiple freeze‐thaw cycles, making them practical to assay.^6^, ^7^ One prospective study of women with breast cancer identified a miRNA signature (miR‐21, miR‐23b, miR‐190, miR‐200b, and miR‐200c) that predicted tumor recurrence and shorter survival.^8^ A panel of serum miRNAs, including miR‐19a, miR‐15, and miR‐181a, correlated with patient tumor burden and decreased after surgical resection.^9^ miR‐331 and miR‐195 accurately discriminate patients with metastatic breast cancer from those with only local disease.^10^ miR‐19a and miR‐205 are higher in patients with Luminal A breast cancer that was chemoresistant to epirubicin and paclitaxel.^11^

There are fewer published studies for miRNA in CMT, particularly as biomarkers in serum or plasma. miR‐126 and miR‐214 both are significantly increased in dogs with mammary carcinoma (along with a number of other malignancies) relative to healthy controls.^12^ Malignant CMT tissues show differential miRNA expression by grade and metastasis, but the proposed miRNAs of interest did not significantly differ in plasma.^13^ A recent in vitro study demonstrated that CMT cells secrete exosomes enriched in miRNAs which could be released into blood, and that the exosomal miRNA pattern is predicted to regulate the estrogen receptor (ESR1), key tumor suppressor PTEN, and other genes relevant to human and canine mammary cancer.^14^ Other in vitro studies have suggested miR‐143 and miR‐138a are dysregulated in some CMT cells, and that miR‐141 plays a role in CMT development by inhibiting tumor suppressor INK4A.^[^ 15, 16 ^]^

There is currently a lack of consensus on the most appropriate normalization strategy for circulating miRNAs.^17^ Normal “reference” genes that are abundant in cells and tissues, such as snoRNAs, are generally absent to minimally detectable in serum. As an alternative approach, some authors recommend absolute quantification through a standard‐curve calibrated to an exogenous spike‐in miRNAs such as cel‐miR‐39.^18^ Others normalize quantitative reverse‐transcription PCR (qPCR) to plasma input volume.^19^ RNA deep‐sequencing (RNAseq) allows relative and absolute quantification based on normalization across millions of all mapped reads.^20^ Digital droplet PCR (dPCR) provides absolute quantification without a normalization gene by measuring tens of thousands of PCR reactions in parallel and assaying against a standard curve for 6‐carboxyfluorescein (FAM) fluorescence.^17^ qPCR and dPCR for miRNAs in lung cancer have high correlation between the assays, with dPCR having lower coefficient of variation and greater reliability.^21^ We determined that the optimal combination of sensitivity and robust results for profiling circulating miRNAs in this cohort was initial target identification by RNAseq validated by dPCR absolute quantification.

Our hypotheses were that (1) the serum miRs would be differentially expressed between healthy dogs and those with CMT with good diagnostic performance, (2) that multiple assay methods (RNAseq and dPCR) would provide similar results, and (3) that these miRNAs would be significantly correlated with tumor grade, lymphatic invasion, inflammatory carcinoma status, and survival time.

## 2 METHODS

### 2.1 Sample groups and tumor pathology

Ten healthy female dogs (5 spayed and 5 intact) were prospectively recruited for the control group and 10 dogs with mammary carcinoma were included in the CMT group. Exclusion criteria for healthy females was any evidence of disease by a veterinarian's physical examination, or abnormalities on CBC or serum biochemistry tests. The 5 healthy intact females varied by stage of estrus at the time of blood collection, and included 3 in estrus, 1 in diestrus, and 1 in anestrus.

Nine of the 10 dogs with mammary carcinoma were enrolled in a previous study on dendritic cell fusion vaccines for CMT; the tumor tissue and serum from all of these dogs were collected before any treatments or interventions.^22^ One of the 10 CMT dogs (MC10) was part of a breeding colony for research dogs and was scheduled for euthanasia because of age and quality of life concerns; a large mammary tumor was discovered before euthanasia, and fresh whole blood, serum, and tumor tissue were collected from this dog immediately postmortem.

Two board‐certified anatomic pathologists blinded to dog identity confirmed the malignant status of the CMT biopsy specimens. Tumors were subtyped histologically, graded, and assessed for the presence or absence of lymphatic/vascular invasion by blinded pathologist blinded to dog identity as previously described.^2^

### 2.2 RNA extraction and miRNA RNAseq

RNA was extracted from 200 μL of serum using the exoRNAeasy midi kit (Qiagen Inc, Valencia, California) according to manufacturer instructions. RNA yield was assessed by Qubit 2.0 RNA fluorometric assay. Extracted RNA was stored at −80°C until being shipped on dry ice to the Genomic Services Laboratory at HudsonAlpha Discovery for deep‐sequencing as previously described.^14^ Briefly, RNA libraries were processed extracted RNA using a NEBNext Small RNA Library Prep Set for Illumina (New England BioLabs Inc, Ipswich, Massachusetts) according to the manufacturer's protocol, with ligation of 3′ and 5′ adapters, reverse transcription (RT) using ProtoScript II RT (New England BioLabs Inc) for 1 hour at 50°C, and PCR amplification by 15 cycles using LongAmp Taq 2X Master Mix (New England BioLabs Inc). Barcoded Illumina primers were added to each sample mix (Illumina, San Diego, California). Post‐PCR material was purified using a QIAquick PCR purification kit (Qiagen Inc) and assessed for yield and purity on a Qubit 2.0 Fluorometer (Invitrogen, Carlsbad, California) and DNA 1000 chip on an Agilent 2100 Bioanalyzer (Applied Biosystems, Carlsbad, California), respectively. Fifty base pair or smaller molecules were selected using 3% dye free agarose gel cassettes on a Pippin Prep instrument (Sage Science Inc, Beverly, Massachusetts). Samples were again assessed post size‐selection on the Qubit 2.0 Fluorometer and DNA High sensitivity chip on Agilent 2100 Bioanalyzer and quantified with the qPCR‐based KAPA Biosystems Library Quantification kit (Kapa Biosystems, Inc, Woburn, Massachusetts). Each library was diluted to a final concentration of 1.25 nM and pooled in equimolar ratios before clustering. Single end (SE) sequencing (50 bp) was performed to generate at least 15 million reads per sample on an Illumina HiSeq2500 sequencer (Illumina Inc).

After processing the sequencing reads from RNA‐seq experiments from each sample was performed as per the HudsonAlpha Discovery unique in‐house pipeline. Briefly, quality control checks on raw sequence data from each sample was performed using FastQC (Babraham Bioinformatics, London, United Kingdom). Raw reads were imported on a commercial data analysis platform (Avadis NGS, Strand Scientifics, California). Adapter trimming was done to remove ligated adapter from 3′ ends of the sequenced reads with only 1 mismatch allowed, poorly aligned 3′ ends were also trimmed. Sequences shorter than 15 nucleotides length were excluded from further analysis. Trimmed reads with low qualities (base quality score less than 30, alignment score less than 95, mapping quality less than 40) were also removed. Filtered reads were then used to extract and count the small RNAs which were annotated using miRNAs from the miRBase release 20 database (http://www.mirbase.org/). Samples were subjected to quantification and active region quantification (Avadis NGS, Strand Scientifics). The quantification operation carries out measurement at both the gene level and at the active region level. Active region quantification considers only reads whose 5′ end matches the 5′ end of the mature miRNA annotation. Samples were then grouped by identifiers and the differential expression of each miRNA was calculated based on the fold change observed between different groups.

### 2.3 Digital droplet RT‐PCR

Seven miRNAs were selected for dPCR validation based on their serum RNAseq expression pattern, previous documentation of relevance to mammary neoplasia in women and dogs in the published literature, and potential target genes: cfa‐miR‐18a, cfa‐miR‐19b, cfa‐miR‐29b, cfa‐miR‐34c, cfa‐miR‐122, cfa‐miR‐125a, and cfa‐miR‐181a. Serum RNA was converted to cDNA using the TaqMan Advanced miRNA cDNA Synthesis Kit (Thermo Fisher Scientific, Waltham, Massachusetts) according to manufacturer instructions. For each 14.5 μL sample, 6 μL of pre‐Amp cDNA template (1 : 10 dilution) was combined with 7.25 μL QuantStudio 3D Digital PCR Master Mix v2 (Thermo Fisher Scientific), 0.725 μL of the appropriate custom ×20 TaqMan Assay (Thermo Fisher Scientific), and 1.53 μL molecular grade water. Reaction tubes were gently vortexed and loaded into an individual QuantStudio 3D Digital PCR Chip v2 (Thermo Fisher Scientific) using the QuantStudio 3D Digital PCR Chip Loader (Thermo Fisher Scientific) according to manufacturer directions. Chips were put into a ProFlex PCR System thermocycler (Thermo Fisher Scientific) and run with the following protocol: 96°C for 10 minutes (1 cycle), 60°C annealing/extension step for 2 minutes followed by 98°C melting step for 30 seconds (39 cycles), and a final stage of 60°C for 2 minutes followed by holding at 10°C. Chips were removed and stored in the dark at room temperature until being read on the QuantStudio 3D Digital PCR Instrument (Thermo Fisher Scientific). Chip data was saved on a USB drive and uploaded to the QuantStudio 3D AnalysisSuite v3.1 (Thermo Fisher Scientific). Absolute quantification was determined through the software algorithm after setting a FAM threshold based on the no template control fluorescence histogram and scatterplot.

### 2.4 Statistical analysis

RNA deep‐sequencing and dPCR results were assessed for normality through visual inspection of QQ plots and Kolmogorov‐Smirnov tests with alpha = .010 using commercially available software (Analyse‐It v2.20, Analyse‐it Software, Ltd, Leeds, United Kingdom). For normally distributed data, a 2‐tailed Student's *t* test was used to compare groups. Non‐parametric data was compared via Wilcoxon‐Mann Whitney tests. Pearson's *r* was tested to evaluate correlation between survival time and circulating miRNAs assessed by RNAseq and dPCR. Receiver operator characteristic (ROC) curves were generated and sensitivity, specificity, and likelihood ratios were calculated. A *P* value of <.05 was considered statistically significant for all hypothesis testing (Benjamini‐Hochberg false discovery rate correction of .05 was applied for RNAseq testing).

## 3 RESULTS

### 3.1 Clinical characteristics

The median age of CMT dogs (10.5 years) was significantly higher than the healthy group (3 years) (*P* = .001). For the CMT group, dog breeds were 2 Labrador Retrievers, and 1 each of the following: Boston Terrier, Bullmastiff, Dachshund, German Shepherd, mixed breed, Rat Terrier, Samoyed, and Shih Tzu. For the healthy control group, all 5 intact females were Labrador Retrievers, whereas the spayed female cohort included 3 mixed breed dogs, 1 Boston Terrier, and 1 Jack Russell Terrier.

Individual dog tumor pathology characteristics are summarized in Table SS1. Of the 10 dogs in the CMT group, 7 had a single tumor and 3 had 2 CMT tumors. The CMT histologic subtypes varied widely. Four dogs had Grade I tumors, 3 had Grade II, and 3 had Grade III. In the 3 dogs that had 2 tumors, both tumors were of the same grade. Six dogs had tumor evidence of lymphatic invasion on their biopsies. Three dogs were treated with only surgical resection of their tumor and no adjunctive treatment (MC1, MC3, and MC8), whereas 1 dog received no treatment as the tumor was found after euthanasia and postmortem examination (MC10). The other 6/10 CMT dogs were treated with standard surgical resection of their tumor(s) followed by gemcitabine chemotherapy and an experimental CMT dendritic cell fusion vaccine.^22^

### 3.2 microRNA profiling by RNAseq

**FIGURE 1:**
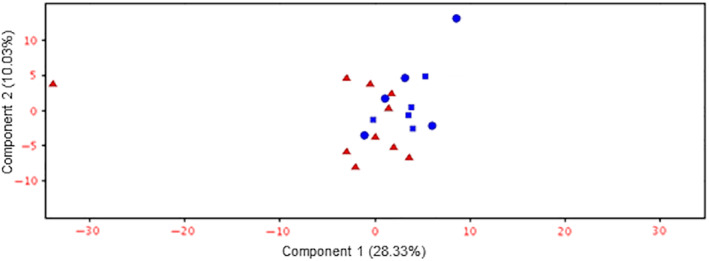
Principal component analysis (PCA) plot for circulating microRNAs. Mammary carcinoma dogs are plotted in red, healthy control dogs are plotted in blue. Square data points represent sexually intact healthy females, whereas circles represent spayed healthy females

The average preamplification RNA concentration from serum was 6.72 ng/μL (range: 20 pg/μL‐129.84 ng/μL; SD: 28.98 ng/μL). RNA fluorograms indicated the samples were high quality and biased towards small RNA populations. Five hundred and eleven total miRNA were detected by RNAseq across the 20 serum RNA samples, with 452 unique miRs (59 miRs were duplicate isoforms from different gene loci). Principal component analysis (PCA) revealed that there was substantial overlap in the overall serum miRNA profile between both intact and spayed healthy females, and partial overlap between the aggregate healthy group and the CMT group (Figure 1).

**FIGURE 2:**
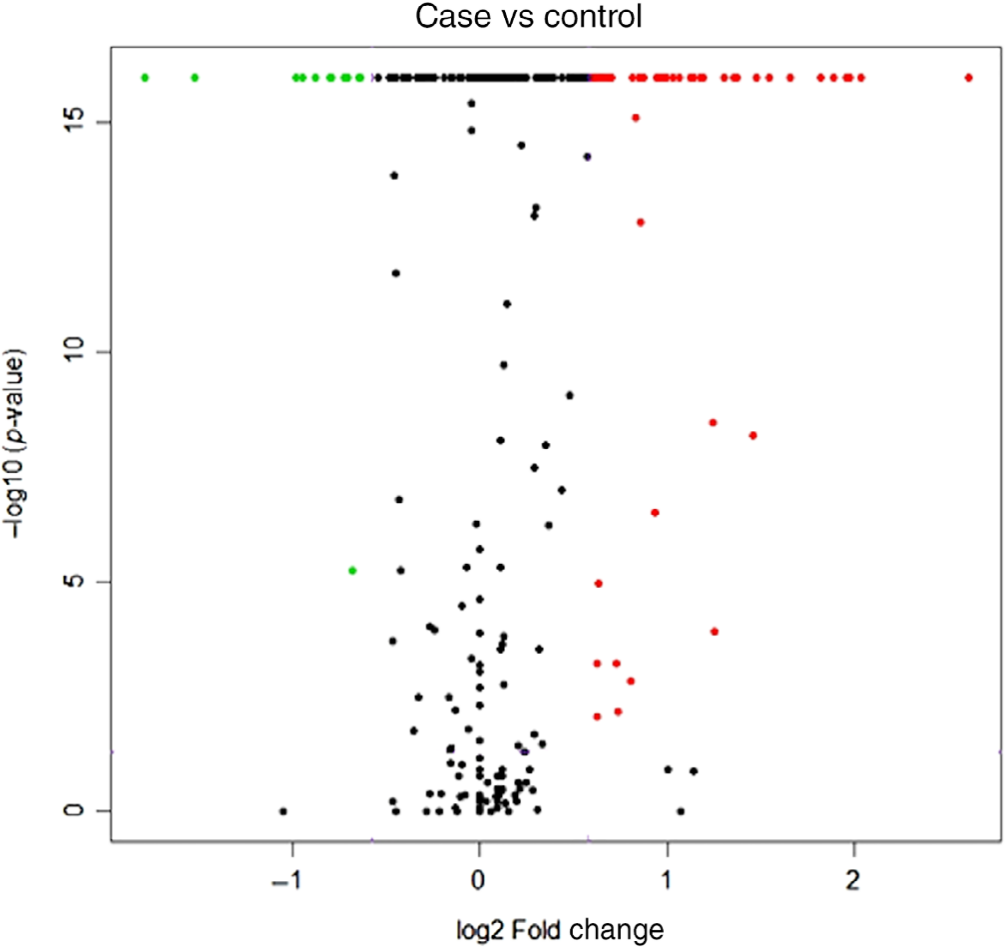
Volcano plot for serum microRNA expression by RNA deep‐sequencing. Red dots in the upper right are significantly upregulated in the canine mammary tumor group by >1.5‐fold, whereas green dots in the upper left are significantly downregulated >1.5‐fold. Black dots were miRs that were expressed with either a <±1.5‐fold difference or not statistically significantly different in expression between groups

Sixty‐five individual miRs were differentially expressed (>±1.5‐fold) and statistically significant between healthy females and those with CMT. The volcano plot in Figure 2 graphically illustrates this differential miRNA expression between groups. Table SS2 shows all significantly differentially expressed miRNAs in CMT samples compared to controls. Some of these upregulated miRs have been previously identified as upregulated in CMT exosomal RNA shed by cultured CMT cells, including miR‐18a, miR‐19b, miR‐29b/c, miR‐34c, miR‐181c, miR‐215, and miR‐345.^14^

### 3.3 Absolute miRNA expression by dPCR

**FIGURE 3:**
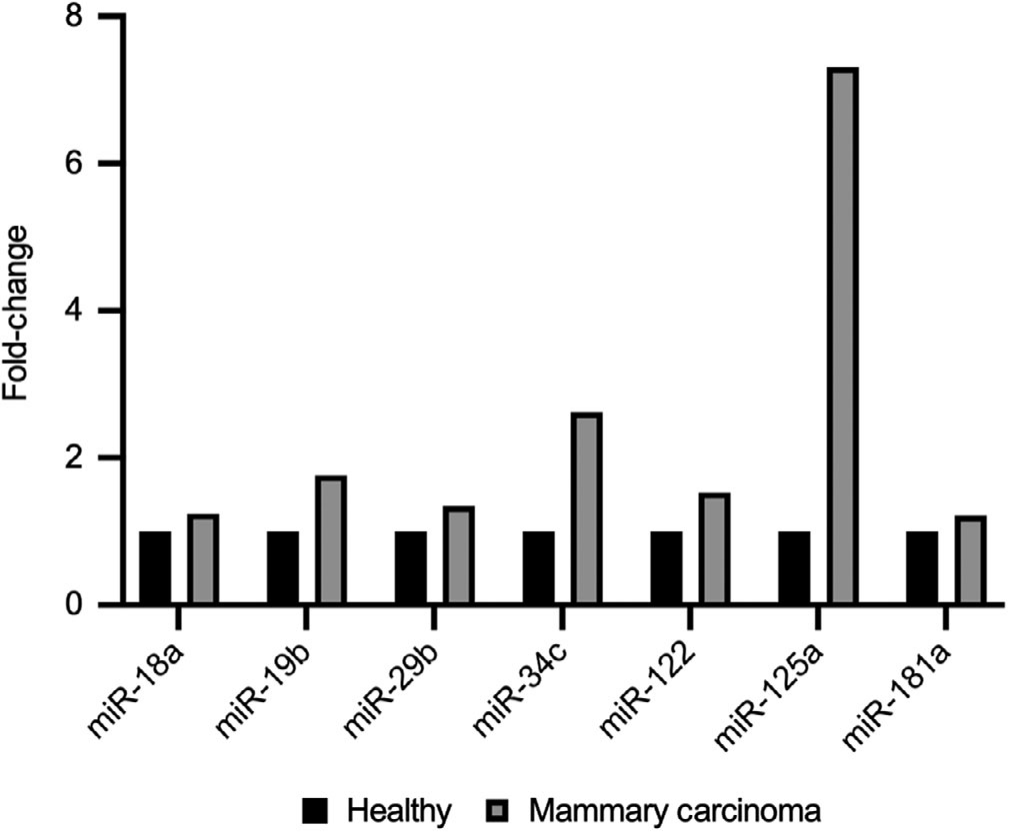
Serum miRNA digital droplet PCR relative expression histogram. Mean expression for each miRNA in copies/μL were compared (dividing mammary carcinoma group values by healthy control group values, setting controls at 1.0 for each target). Gray bars are fold‐change for the mammary carcinoma group relative to the healthy controls (black bars)

**FIGURE 4:**
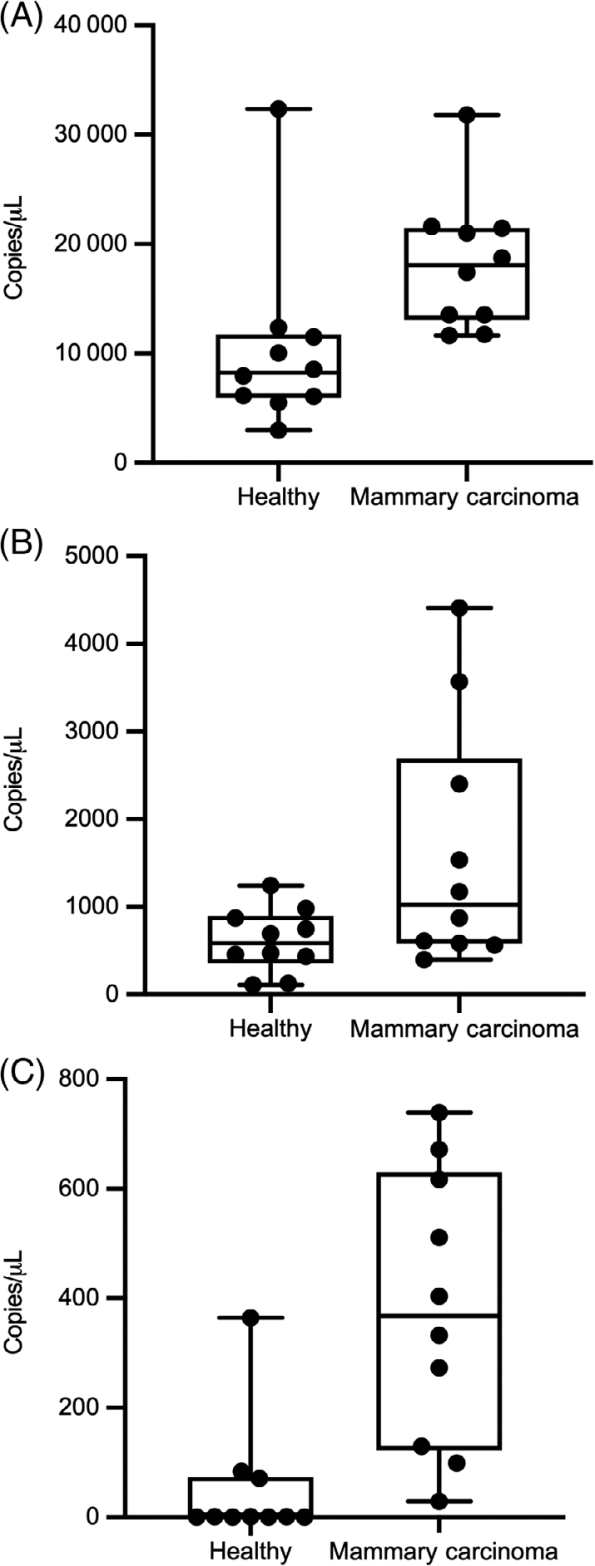
Box and whisker plots for absolute miRNA expression by digital droplet PCR between mammary carcinoma dogs and healthy dogs (in copies per μL). Bold horizontal lines are median values. A, miR‐19b; B, miR‐34c; C, miR‐125a

Absolute miRNA expression data for miR‐18a, miR‐19b, miR‐29b, miR‐34c, miR‐122, miR‐125a, and miR‐181a are summarized in Table 1. miR‐18a, miR‐19b, and miR‐181a were the most abundantly expressed miRs in the set tested by dPCR, with others having lower absolute expression. miR‐34c and miR‐125a had the largest magnitude relative fold‐change between the mammary carcinoma group and healthy control group (Figure 3). Both miR‐19b and miR‐125a were significantly higher in the mammary carcinoma group than among healthy control dogs by dPCR (Figure 4A,C). miR‐34c was substantially higher among dogs with mammary carcinoma than healthy subjects, although this difference narrowly missed statistical significance (Figure 4B, Table 2). One dog in the healthy control group (subject HS3) was an outlier with extremely high miR‐19b expression (32 364 copies/μL). Clinical follow‐up on this dog revealed that within 1 year of this sample collection it developed widespread pulmonary metastasis from an unknown primary cancer and died shortly thereafter.

**TABLE 1:** Serum miRNA absolute expression by digital droplet PCR (in copies per μL)

microRNA	Mammary carcinoma group				Healthy control group				*P* value
	Mean	Median	SD	Range	Mean	Median	SD	Range	
miR‐18a	33 397.4	28 679.0	16 977.7	10 720.0‐62 271.0	26 945.1	24 270.0	16 459.0	7381.4‐60 453.0	.40
miR‐19b*	18 256.3	18 073.0	6186.7	11 635.0‐31 825.0	10 357.4	8249.4	8249.4	2993.9‐32 364.0	**.003**
miR‐29b	3059.9	2921.0	1156.9	1707.4‐5336.3	2276.1	1362.1	2555.8	284.95‐8801.2	.39
miR‐34c	1611.6	1023.9	1400.6	396.2‐4410.4	614.0	584.3	363.9	110.22‐1242.3	.08
miR‐122	284.6	254.5	181.3	48.9‐635.6	186.0	132.8	164.6	36.7‐559.0	.22
miR‐125a*	380.6	368.0	250.8	29.2‐739.1	52.1	0.7	114.2	0.0‐364.1	**<.001**
miR‐181a	12 503.2	10 364.5	4765.0	8611.9‐23 296.0	10 211.4	8396.3	5957.0	3564.7‐19 808.0	.36

*Note: P* values are for univariate statistical comparison between groups. An asterisk (*) indicates nonparametric data, medians compared by Wilcoxon rank‐sum test. All other variables are normally distributed and means compared by 2‐tailed Student's *t* test. *P* < .05 was considered statistically significant (statistically significant *P* values highlighted in bold).

**TABLE 2:** Comparison of digital droplet PCR (dPCR) and RNA deep‐sequencing (RNAseq) results for select microRNAs

microRNA	RNAseq		dPCR	
	Fold‐change	*P* value	Fold‐change	*P* value
cfa‐miR‐18a	1.94	**.000**	1.24	.40
cfa‐miR‐19b	3.15	**.000**	1.76	**.003**
cfa‐miR‐29b	2.78	**.000**	1.34	.39
cfa‐miR‐34c	6.07	**.000**	2.62	.075
cfa‐miR‐122	−2.87	**.000**	1.53	.22
cfa‐miR‐125a	−3.46	**.000**	7.31	**<.001**
cfa‐miR‐181a	1.02	.500	1.22	.36

*Note:* Relative fold change in the mammary carcinoma group compared to the healthy control group. Significant *P* values are bolded.

RNA deep‐sequencing and dPCR assays were compared by assessing miRNA fold‐change and statistical significance between the mammary carcinoma and healthy control groups, and this data is summarized in Table 2. Two of 7 miRNAs were significantly different by both methodologies (miR‐19b and miR‐125a). Results between the assays were largely similar in direction of fold‐change, with the notable exception of miR‐125a and miR‐122, which were both increased by dPCR despite being downregulated according to RNAseq. Six of 7 miRNAs had less‐extreme fold‐change by dPCR than RNAseq (with the exception of miR‐125a). miR‐181a was abundantly expressed in both carcinoma and control cohorts, but did not differ statistically between groups by either RNAseq or dPCR.

### 3.4 Diagnostic performance

**FIGURE 5:**
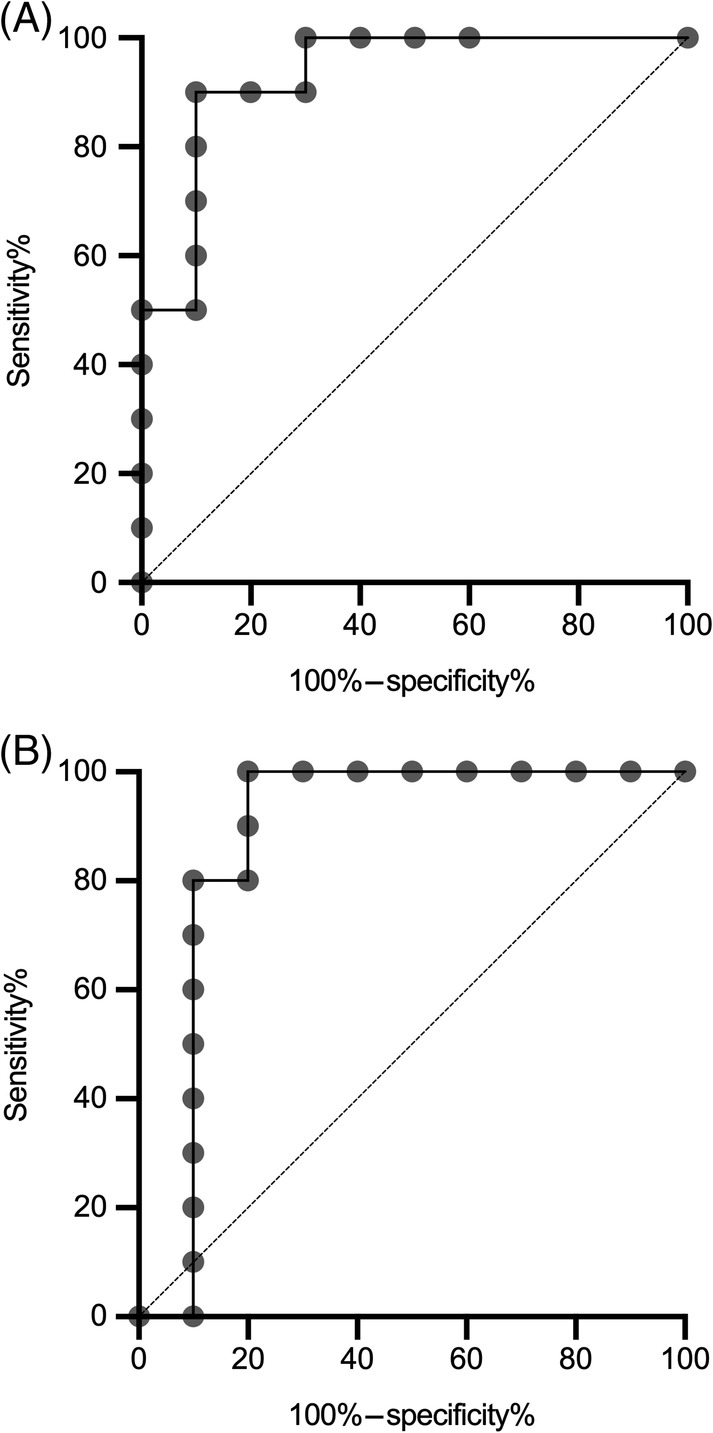
Receiver operator characteristic‐area under the curve (AUC) plots for A, miR‐125a, and B, miR‐19b. The light gray line indicates an AUC of 0.50 and no ability to discriminate diseases beyond chance

To evaluate the ability of these miRNAs to discriminate clinical cases from control subjects, ROC plots were generated. The highest ROC area under the curve (AUC) was miR‐125a at 0.930 (Figure 5A), indicating excellent ability to discriminate between dogs with mammary carcinoma and healthy controls in this population. miR‐19b also had a high ROC‐AUC at 0.880 (Figure 5B). When excluding the outlier healthy control HS3 because of the possibility of occult neoplasia, the AUC‐ROC for miR‐19b increased to 0.978, which would indicate near‐perfect ability to discriminate mammary tumor‐bearing dogs from dogs without neoplasia. All other miRNAs had fair to poor ROC‐AUC (Table 3).

**TABLE 3:** Receiver operator characteristic analysis for 7 miRNA

microRNA	AUC	95% CI	SE
cfa‐miR‐18a	0.630	0.372‐0.888	0.132
cfa‐miR‐19b	0.880	0.683‐1.077	0.101
cfa‐miR‐29b	0.790	0.542‐1.038	0.126
cfa‐miR‐34c	0.740	0.513‐0.967	0.116
cfa‐miR‐122	0.690	0.430‐0.950	0.132
cfa‐miR‐125a	0.930	0.816‐1.044	0.058
cfa‐miR‐181a	0.650	0.380‐0.920	0.138

Because of suitable biomarker characteristics for miR‐19b (high absolute expression, strong upregulation by the CMT group by RNAseq and dPCR, robust ROC‐AUC), additional diagnostic test parameters were calculated for this miRNA. The diagnostic sensitivity and specificity of miR‐19b varied by the selected cutoff, and whether dog HS3 was included or not. At 11 600 copies/μL and including HS3, miR‐19b had a sensitivity of 100%, a specificity of 80%, and a positive likelihood ratio of 5.0 (95% CI: 1.96‐17.64). At a cutoff of 13 000 copies/μL, the sensitivity decreased to 80% whereas specificity increased to 90%; the positive likelihood ratio increased to 8.0 (95% CI: 1.82‐45.48), whereas the negative likelihood ratio was 0.22 (95% CI: 0.06‐0.61). Excluding HS3 at 11 600 copies/μL, sensitivity was 100%, specificity 88.9%, and the positive likelihood ratio was 9.0 (95% CI: 2.30‐50.27). Excluding HS3 at 13 000 copies/μL, sensitivity was 80%, specificity was 100%, and negative likelihood ratio was 0.20 (95% CI: 0.06‐0.51).

### 3.5 miRNA association with histopathologic characteristics

**FIGURE 6:**
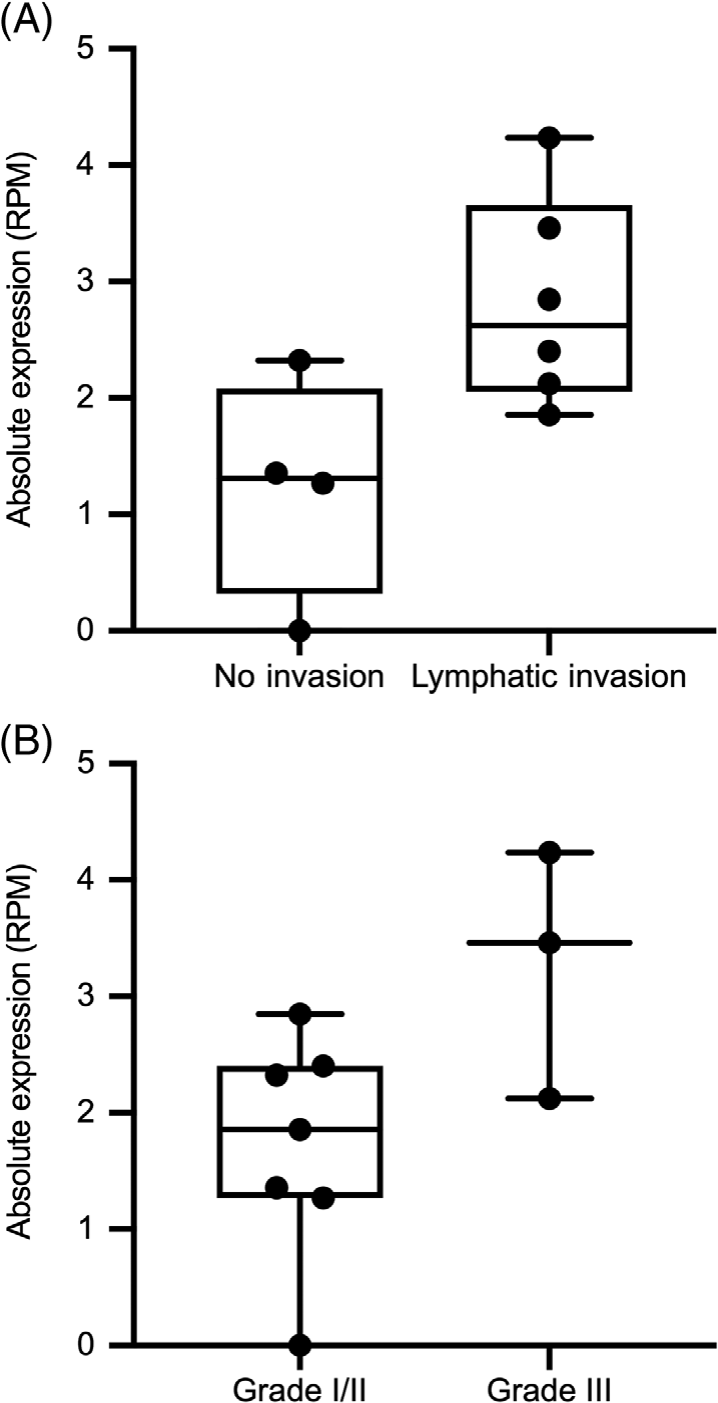
Box and whisker plots absolute miR‐18a expression by RNA deep‐sequencing based on tumor prognostic factors (in reads per million, RPM). Bold horizontal lines are median values. A, canine mammary tumor (CMT) dogs with and without lymphatic invasion. B, CMT dogs with high grade tumors (Grade III) versus Grade I/II tumors

Circulating miRNA expression for these 7 targets was compared between mammary carcinoma dogs with Grade III tumors (high grade) versus Grade I/II tumors (low grade), as well between mammary carcinoma dogs with and without metastasis. miR‐18a by RNAseq was significantly higher in the group with lymphatic invasion than without (2.82 versus 1.23 RPM, *P* = .03) (Figure 6A). miR‐18a was not higher by RNAseq for the 3 dogs with Grade III tumors compared to all others (3.27 versus 1.72 RPM; *P* = .05) (Figure 6B). No other serum miRNAs analyzed by RNAseq or dPCR were significantly different between metastasis or grade groups. There was also no significant difference between serum miRNAs by RNAseq or dPCR between CMT dogs that developed inflammatory mammary carcinoma from those that did not.

### 3.6 Circulating miRNA association with survival

Survival times from time of surgical resection to spontaneous death or euthanasia were available for 8 of 10 dogs in the CMT group. There were no statistically significant correlations between any of the 7 circulating miRNAs by RNAseq or dPCR and survival time in days (Table 4).

**TABLE 4:** Correlation between circulating miRNA expression and survival time

Comparison	Pearson's *r*	*P* value
Serum miR‐18a (RNAseq) versus survival (days)	0.011	.98
Serum miR‐18a (dPCR) versus survival (days)	0.387	.34
Serum miR‐19b (RNAseq) versus survival (days)	−0.103	.81
Serum miR‐19b (dPCR) versus survival (days)	−0.110	.80
Serum miR‐29b (RNAseq) versus survival (days)	−0.497	.21
Serum miR‐29b (dPCR) versus survival (days)	0.186	.66
Serum miR‐34c (RNAseq) versus survival (days)	−0.498	.21
Serum miR‐34c (dPCR) versus survival (days)	−0.530	.18
Serum miR‐122 (RNAseq) versus survival (days)	−0.017	.97
Serum miR‐122 (dPCR) versus survival (days)	−0.293	.48
Serum miR‐125a (RNAseq) versus survival (days)	−0.673	.07
Serum miR‐125a (dPCR) versus survival (days)	−0.314	.45
Serum miR‐181a (RNAseq) versus survival (days)	0.131	.76
Serum miR‐181a (dPCR) versus survival (days)	−0.186	.66

Abbreviations: dPCR, digital droplet PCR; RNAseq, RNA deep‐sequencing.

## 4 DISCUSSION

Our study demonstrates that serum from dogs with mammary carcinoma is enriched with hundreds of circulating miRNAs. While the overall expression pattern between dogs with malignant CMT and healthy controls had substantial overlap based on PCA, a number of individual miRNAs were significantly upregulated or downregulated in the CMT group. Previous in vitro research on CMT exosomes reveals a number of these, including miR‐18a, miR‐19b, miR‐29b/c, miR‐34c, miR‐181c, miR‐215, and miR‐345 that are predicted to target a number of important genes and pathways relevant to mammary tumorigenesis, such as ESR1 and the tumor suppressor PTEN.^14^ Of these miRNAs, miR‐29b is upregulated in malignant CMT versus normal mammary tissue.^23^ Circulating miR‐126 and miR‐214 are both increased in dogs with a variety of tumors, including mammary malignancy, however neither was between those significantly or differentially expressed between CMT and healthy dogs in this dataset.^12^

miR‐19b was strongly and significantly upregulated in the CMT group by both RNAseq and dPCR. Furthermore, ROC‐AUC and sensitivity/specificity analysis indicated this particular miRNA had good ability to differentiate between the 2 groups. Although this is a small cohort, and neither animals with nonneoplastic mammary disease (ie, mastitis) nor subjects with nonmammary neoplasms were included, this suggests miR‐19b is worth further investigation as a potential biomarker for mammary carcinoma in dogs. This agrees with prior studies that show circulating miR‐19a (closely related to miR‐19b) has prognostic significance in women with breast cancer.^9^, ^11^

Interestingly, 1 dog in the healthy spayed female control cohort (HS3) had extremely high miR‐19b expression and went on to develop widespread metastasis from an unidentified primary cancer within 12 months of sample collection. While this dog did not show any outward evidence of occult malignancy on physical examination or laboratory testing, diagnostic imaging was not an inclusion criterion for the healthy controls and it is possible this dog had a small, unidentified tumor at the time of blood collection. The conserved miR‐19 family, which includes the highly similar miR‐19a and miR‐19b, has been proposed as a biomarker for multiple cancers in human oncology, including women with breast cancer. miR‐19a was significantly associated with chemoresistance in women with Luminal A breast cancer, and levels were greater than 2‐fold higher in chemoresistant compared to chemosensitive human breast cancer cases.^11^ However, the lack of either postmortem examination or tumor histopathology and immunohistochemistry limited the inferences that could be drawn from this outlier. Because of the possibility that this dog was actually not a false positive, but rather had early malignancy that had not yet manifested itself obviously, AUC‐ROC, sensitivity, specificity, and likelihood ratio analysis was run with and without this dog included, and results for both were presented.

miR‐125a was significantly different between CMT and healthy groups by both RNAseq and dPCR, and also showed diagnostic promise based on AUC‐ROC. This could fit with a previous study that showed differential tissue miR‐125a expression between dogs with metastatic carcinoma and benign adenoma.^24^ However, the difference in fold‐change between assays which were downregulated by RNAseq and upregulated by dPCR raised concerns about the reliability of this miRNA as a biomarker and was not investigated in greater detail. RNAseq and dPCR generate relative and absolute quantification of genes through different methodologies, which could explain the mismatch. One potential source of discrepancy is the library preparation process required by RNAseq, but not dPCR.^20^

To investigate the potential for serum miRNAs to predict relevant prognostic factors, 7 candidate circulating miRNAs were compared among CMT subgroups for those with inflammatory carcinoma, Grade III versus Grade I/II tumors and presence or absence of lymphatic invasion, as these are well‐known histopathologic features that impact prognosis.^2^, ^3^, ^4^ Serum miR‐18a concentration by RNAseq was significantly higher in dogs with histologic evidence of tumor lymphatic invasion. Thus, circulating miR‐18a merits further evaluation as a possible predictive marker of metastasis and possible high‐grade status in CMT tumors in larger prospective longitudinal studies. Additionally, given that serum miR‐18a expression was significantly different by RNAseq but not dPCR, the impact of assay methodology on quantification between RNAseq, dPCR, and qPCR should be investigated, and a consensus gold standard should be established.

No other serum miRNA was significantly different between lymphatic invasion, inflammatory carcinoma, or high‐grade status by RNAseq or dPCR methods. Regardless, these differentially expressed circulating miRNAs should still be evaluated in a large prospective cohort. For many of these histopathologic parameters, the group sizes were very small (n = 3 for Grade III tumors and inflammatory carcinoma), which likely decreased the statistical power to detect any small but real changes. Finally, the number of histologic subtypes was too variable to compare serum miRNA expression statistically, but subtypes such as micropapillary and cribriform mammary carcinomas have a worse prognosis, so a larger sample size might be able to study the relationship of miRs to these rarer variants.

There was no statistically significant correlation between circulating miRNAs and survival times for the 8/10 dogs where that data was available. However, it is difficult to draw definitive conclusions from this population for several reasons. First, the sample size was small and did not include 2 of the CMT dogs. Second, the treatment protocols and clinical course varied widely between dogs in the cohort. Dogs MC2, MC5, and MC6 were treated with an experimental dendritic cell fusion vaccine and gemcitabine following surgical resection of the CMT.^22^ Three additional dogs (MC4, MC7, and MC9), received this same protocol, but developed inflammatory mammary carcinoma and died shortly thereafter. Two dogs received only surgical resection of their tumor with no follow‐up chemotherapy, radiation treatment, or other adjunctive treatment. A prospective study with a far larger sample size is necessary to determine what relationship, if any, there is between these circulating miRNA and response to treatment, progression free survival, risk of relapse, and overall survival.

Our study has a number of limitations. First, the small sample size might have been underpowered to detect modest but real group differences, especially for dPCR. Notably, absolute expression for miR‐34c was prominently upregulated in the mammary carcinoma group, but the *P* value was slightly above the alpha .05 boundary of statistical significance for dPCR. However, despite the modest number of biological replicates, RNAseq identified millions of small RNA reads, many of which were differentially expressed. The risk of false positives detected by RNAseq simultaneously analyzing hundreds of miRNAs was mitigated statistically through the Benjamini‐Hochberg correction procedure and technically by validation with a different quantification method (dPCR).^25^ In addition, both the mammary carcinoma and healthy control group subjects were robust, with the former having a variety of tumors of different histologic type and grade, and the latter including both OHE dogs and bitches in various stages of estrus. The diversity in histopathologic characteristics is especially important, and 2 important prognostic factors included high‐grade tumors (Grade III) and tumors with lymphatic invasion.^2^ Second, this population did not include subjects with nonmalignant mammary pathology such as mastitis or benign mammary tumors. This could be relevant as research evaluating miRNA in cow and porcine milk has identified particular miRNA signature that increase with mastitis, including miR‐21, miR‐146a, miR‐155, miR‐222, and miR‐383.^26^ Fortunately, these miRNAs are not among the most relevant potential biomarkers identified in this dataset.

Overall, our study identified a number of circulating miRNAs that were significantly over‐expressed, under‐expressed, or both by dogs diagnosed with mammary carcinoma relative to healthy controls. Some of these, such as miR‐19b, have good diagnostic test performance, and could represent candidate biomarkers for CMT. Further prospective studies on a larger cohort of CMT dogs are warranted to evaluate the diagnostic utility of circulating miR‐19b. Future research should directly evaluate the impact of miRNA on ER and PR expression through in vitro studies that transfect relevant miRNA mimics and inhibitors to CMT cell lines and measure resulting mRNA and protein expression changes by qPCR and western blot/flow cytometry, respectively.

## OFF‐LABEL ANTIMICROBIAL DECLARATION

Authors declare no off‐label use of antimicrobials.

## CONFLICT OF INTEREST DECLARATION

Authors declare no conflict of interest.

## INSTITUTIONAL ANIMAL CARE AND USE COMMITTEE (IACUC) OR OTHER APPROVAL DECLARATION

This research project was approved by, and conducted under the oversight of, the Auburn University College of Veterinary Medicine Clinical Research Review Committee and the Auburn University IACUC in AAALAC approved animal care facilities under IACUC PRN 2016‐2834 and IACUC PRN 2015‐2688.

## HUMAN ETHICS APPROVAL DECLARATION

Authors declare human ethics approval was not needed for our study.

## Supporting information


**Table S1** CMT group tumor histopathologic subtype, grade, lymphatic invasion, and survival time.

**Table S2** Differentially expressed microRNA, fold‐change, and *p*‐values by RNAseq.
