# Supplementary material for: Circulating microRNA as biomarkers of canine mammary carcinoma in dogs
Source: J Vet Intern Med. 2020 Apr 27;34(3):1282–90. doi: 10.1111/jvim.15764 (PMC7255679; doi:10.1111/jvim.15764)
Supplement: Supplementary file 1 — Table S1 CMT group tumor histopathologic subtype, grade, lymphatic invasion, and survival time. [file JVIM-34-1282-s001.pdf]

Supplemental Table 1: CMT group tumor histopathologic subtype, grade, lymphatic invasion, and survival time

Case	Histologic subtype	Grade	Lymphatic invasion	Inflammatory BC?	Survival (Days)
MC1	Carcinoma-mixed type	Grade I	Yes--Lymphatic	No	183
MC2	Carcinoma--anaplastic	Grade III	Yes--Lymphatic	No	611
MC3	Carcinoma--cystic papillary; carcinoma--	Grade I (both)	No	No	63
MC4	Carcinoma-micropapillary invasive	Grade II	Yes--Lymphatic	Yes	83
MC5	Carcinoma-spindle variant	Grade II	No	No	427
MC6	Carcinoma-mixed type; Carcinoma comp	Grade I (both)	No	No	636
MC7	Carcinoma--solid; Carcinoma-micropapil	Grade II (both)	Yes--Lymphatics; LN+	Yes	85
MC8	Carcinoma in situ	Grade I	No	No	n/a
MC9	Carcinoma-anaplastic	Grade III	Yes--Lymphatics; LN+	Yes	42
MC10	Carcinoma--tubulopapillary/solid	Grade III	Yes--Lymphatics	No	n/a
