# Supplementary material for: Circulating microRNA as biomarkers of canine mammary carcinoma in dogs
Source: J Vet Intern Med. 2020 Apr 27;34(3):1282–90. doi: 10.1111/jvim.15764 (PMC7255679; doi:10.1111/jvim.15764)
Supplement: Supplementary file 2 — Table S2 Differentially expressed microRNA, fold‐change, and p‐values by RNAseq. [file JVIM-34-1282-s002.pdf]

microRNA	Fold-change	p-value
cfa-miR-34c	6.08	0.00E+00
cfa-miR-135a-5p	4.08	0.00E+00
cfa-miR-199	3.93	0.00E+00
cfa-miR-182	3.93	0.00E+00
cfa-miR-199	3.88	0.00E+00
cfa-miR-199	3.88	0.00E+00
cfa-miR-30b	3.69	0.00E+00
cfa-miR-135a-5p	3.51	0.00E+00
cfa-miR-19b	3.15	0.00E+00
cfa-miR-19b	3.14	0.00E+00
cfa-miR-23a	2.92	0.00E+00
cfa-miR-29b	2.78	0.00E+00
cfa-miR-29b	2.78	0.00E+00
cfa-miR-504	2.74	2.50E-09
cfa-miR-421	2.59	0.00E+00
cfa-miR-223	2.56	0.00E+00
cfa-miR-374b	2.47	0.00E+00
cfa-miR-215	2.38	4.83E-05
cfa-miR-345	2.36	1.28E-09
cfa-miR-502	2.28	0.00E+00
cfa-miR-106b	2.25	0.00E+00
cfa-miR-1842	2.21	0.00E+00
cfa-miR-107	2.19	0.00E+00
cfa-miR-331	2.17	0.00E+00
cfa-miR-15b	2.09	0.00E+00
cfa-miR-1839	2.04	0.00E+00
cfa-miR-23b	2.03	0.00E+00
cfa-miR-9	1.99	0.00E+00
cfa-miR-9	1.99	0.00E+00
cfa-miR-9	1.99	0.00E+00
cfa-miR-103	1.97	0.00E+00
cfa-miR-103	1.95	0.00E+00
cfa-miR-18a	1.94	0.00E+00
cfa-miR-374a	1.93	0.00E+00
cfa-miR-339	1.92	0.00E+00
cfa-miR-200c	1.91	1.17E-07
cfa-miR-365	1.83	0.00E+00
cfa-miR-365	1.83	0.00E+00
cfa-miR-183	1.81	5.38E-14
cfa-miR-20a	1.8	0.00E+00

cfa-miR-362	1.8	0.00E+00
cfa-miR-8884	1.79	0.00E+00
cfa-miR-190b	1.78	2.84E-16
cfa-miR-221	1.76	0.00E+00
cfa-miR-425	1.75	0.00E+00
cfa-miR-1843	1.74	6.19E-04
cfa-miR-203	1.66	2.80E-03
cfa-miR-127	1.66	2.47E-04
cfa-miR-16	1.62	0.00E+00
cfa-miR-181c	1.62	0.00E+00
cfa-miR-132	1.61	0.00E+00
cfa-miR-15a	1.61	0.00E+00
cfa-miR-350	1.59	0.00E+00
cfa-miR-197	1.58	0.00E+00
cfa-miR-218	1.56	0.00E+00
cfa-miR-218	1.56	0.00E+00
cfa-miR-301b	1.55	4.29E-06
cfa-miR-133a	1.54	3.62E-03
cfa-miR-133c	1.54	3.62E-03
cfa-miR-8865	1.54	2.58E-04
cfa-miR-551b	1.53	0.00E+00
cfa-miR-16	1.52	0.00E+00
cfa-miR-29c	1.52	0.00E+00
cfa-miR-29c	1.52	0.00E+00
cfa-miR-32	1.52	0.00E+00
cfa-let-7d	-1.56	0.00E+00
cfa-miR-155	-1.57	0.00E+00
cfa-miR-139	-1.6	2.22E-06
cfa-miR-486	-1.64	0.00E+00
cfa-miR-423a	-1.65	0.00E+00
cfa-miR-99b	-1.73	0.00E+00
cfa-miR-10a	-1.74	0.00E+00
cfa-miR-6529	-1.84	0.00E+00
cfa-let-7b	-1.93	0.00E+00
cfa-miR-375	-1.97	0.00E+00
cfa-miR-122	-2.88	0.00E+00
cfa-miR-125a	-3.46	0.00E+00
